# AKT2 is the predominant AKT isoform expressed in human skeletal muscle

**DOI:** 10.14814/phy2.13652

**Published:** 2018-03-29

**Authors:** Ronald W. Matheny, Alyssa V. Geddis, Mary N. Abdalla, Luis A. Leandry, Michael Ford, Holly L. McClung, Stefan M. Pasiakos

**Affiliations:** ^1^ Military Performance Division US Army Research Institute of Environmental Medicine Natick Massachusetts; ^2^ MS Bioworks Ann Arbor, Michigan; ^3^ Military Nutrition Division US Army Research Institute of Environmental Medicine Natick Massachusetts

**Keywords:** AKT, differentiation, myoblast, skeletal muscle

## Abstract

Skeletal muscle physiology and metabolism are regulated by complex networks of intracellular signaling pathways. Among many of these pathways, the protein kinase AKT plays a prominent role. While three AKT isoforms have been identified (AKT1, AKT2, and AKT3), surprisingly little is known regarding isoform‐specific expression of AKT in human skeletal muscle. To address this, we examined the expressions of each AKT isoform in muscle biopsy samples collected from the vastus lateralis of healthy male adults at rest. In muscle, *AKT2* was the most highly expressed AKT transcript, exhibiting a 15.4‐fold increase over *AKT1* and *AKT3* transcripts. Next, the abundance of AKT protein isoforms was determined using antibody immunoprecipitation followed by Liquid Chromatography‐Parallel Reaction Monitoring/Mass Spectrometry. Immunoprecipitation was performed using either mouse or rabbit pan AKT antibodies that were immunoreactive with all three AKT isoforms. We found that AKT2 was the most abundant AKT isoform in human skeletal muscle (4.2‐fold greater than AKT1 using the rabbit antibody and 1.6‐fold greater than AKT1 using the mouse antibody). AKT3 was virtually undetectable. Next, cultured primary human myoblasts were virally‐transduced with cDNAs encoding either wild‐type (WT) or kinase‐inactive *AKT1* (AKT1‐K179M) or *AKT2* (AKT2‐K181M) and allowed to terminally differentiate. Myotubes expressing WT‐AKT1 or WT‐AKT2 showed enhanced fusion compared to control myotubes, while myotubes expressing AKT1‐K179M showed a 14% reduction in fusion. Myotubes expressing AKT2‐K181M displayed 63% decreased fusion compared to control. Together, these data identify AKT2 as the most highly‐expressed AKT isoform in human skeletal muscle and as the principal AKT isoform regulating human myoblast differentiation.

## Introduction

AKT is a serine/threonine kinase that mediates a number of intracellular processes including proliferation, survival, and metabolism (Manning and Cantley 2007). Three AKT isoforms have been identified in mammals (AKT1, AKT2, and AKT3), and there exist distinct and redundant functions for each (Cho et al. 2001a,b; Easton et al. 2005). In skeletal muscle, AKT1 has been shown to regulate proliferation, growth, and hypertrophy (Izumiya et al. 2008; Wilson and Rotwein 2007), while AKT2 has been shown to regulate glucose uptake, fatty acid transport, and glycogen synthase activity (Jain et al. 2015; Hussain et al. 2011; Friedrichsen et al. 2013). Both Akt1 and Akt2 contribute to murine myoblast differentiation, although Akt1 has been shown to play a more prominent role than Akt2 (Gardner et al. 2012). Little is known regarding the role of AKT3 in skeletal muscle. While a great deal of our knowledge regarding the functions of the three AKT isoforms in muscle has been derived from the use of cell and animal models, much less is known regarding the expression of AKT in human skeletal muscle. Given this, we performed a series of experiments designed to determine the abundances of each AKT isoform in human skeletal muscle and to identify which AKT isoform is the predominant AKT involved in human myoblast differentiation.

## Materials and Methods

### Materials and reagents

Low glucose Dulbecco's modified Eagle's medium (#11885‐092), horse serum (#26050‐70), BacMam enhancer solution (#PV5835), primary human skeletal myoblasts (#A1140), recombinant full‐length AKT1 (#P2999), recombinant full‐length AKT2 (#PV3184), and recombinant full‐length AKT3 (#PV3185), were purchased from Life Technologies (Carlsbad, CA). Antibodies for AKT1 (C73H10; #2938), AKT2 (D6G4; #3063), AKT3 (E2B6R; #14293), AKT (Pan) (C67E7) (rabbit monoclonal; #4691), AKT (Pan) (40D4) (mouse monoclonal; #2920), and Protein A HRP (#12291S), were purchased from Cell Signaling Technologies (Danvers, MA). Normal rabbit IgG (#sc‐2027) and normal mouse IgG (#sc‐2025) were purchased from Santa Cruz Biotechnology (Santa Cruz, CA).

### Subjects and human skeletal muscle

Human skeletal muscle samples were obtained from a previously published study registered at clinicaltrials.gov as NCT017366924. The investigators adhered to U.S. Army Regulation 70–25 and U.S. Army Medical Research and Material Command regulation 70–25 on the participation of volunteers in research. All study procedures in human volunteers were approved by the Institutional Review Board at the U.S. Army Research Institute of Environmental Medicine, Natick, MA. Muscle biopsy samples were obtained at rest from the vastus lateralis of healthy adult men (age, years: 23 ± 5.3; weight, kg: 72.4 ± 12.3; height, cm: 178.3 ± 5.4; body fat, % (via DEXA): 18.7 ± 3.1) after obtaining informed, written consent (Pasiakos et al. 2011). Muscle samples were collected under local anesthesia (1% lidocaine) using a 5 mm Bergstrom needle (Evans et al. 1982), cleaned of visible fat, blotted for excess blood, then frozen in liquid nitrogen and stored at −80°C until analyzed.

### Cell Culture, baculoviruses, and transductions

Culture and differentiation of primary human skeletal myoblasts was performed as described previously (Matheny et al. 2015). Open reading frame clones encoding human AKT1 and AKT2 in the pENTR221 vector were purchased from Life Technologies. The AKT1 K179M and AKT2 K181M point mutations were introduced by standard PCR‐based site‐directed mutagenesis. Generation of bacmid DNA, SF9 insect cell‐mediated virus production, and viral transductions of human myoblasts were performed as described (Matheny et al. 2015).

### RNA isolation, cDNA synthesis, and real‐time PCR

RNA isolation and cDNA synthesis were performed as described previously (Matheny et al. 2012) using Trizol reagent (Life Technologies). Real time PCR was performed as previously described (Matheny et al. 2015), normalizing to *HPRT1* gene expression. Primers/probes for real‐time PCR were purchased from Life Technologies: *AKT1* (Hs00920503_m1), *AKT2* (Hs01086102_m1), *AKT3* (Hs00987350_m1), *HPRT1* (Hs02800695_m1).

### Protein extraction, immunoprecipitation, and immunoblotting

Extraction of protein from muscle samples was performed as described (Matheny et al. 2015), and protein concentrations were determined by the method of Bradford (Bradford 1976). Immunoprecipitations and western immunoblotting were performed exactly as described previously (Matheny et al. 2015), using Protein A HRP as the secondary antibody. Immunoprecipitations were performed using 250 *μ*g protein lysate and 1:5 (v/v) protein A/G magnetic beads (Life Technologies). For analysis of AKT synthetic peptides, 50 ng of full‐length recombinant peptide (in water) was boiled with 5X Laemmli sample buffer for 5‐min before subjecting samples to SDS‐PAGE and western immunoblotting.

### Liquid chromatography‐parallel reaction monitoring/mass spectrometry (LC‐PRM/MS)

Immunoprecipitated samples were eluted from the magnetic beads from the immunoprecipitation by heating in 60 *μ*L 1.5x NuPAGE LDS Sample Buffer (ThermoFisher Scientific) at 100°C for 15 min. Half of the elution was processed by SDS‐PAGE using a 10% Bis‐Tris NuPAGE gel (Life Technologies) with the MES buffer system; the gel was run approximately 1 cm. The mobility region was excised and processed by in‐gel digestion with trypsin using a ProGest robot (Digilab). Excised gel bands were first washed with 25 mmol/L ammonium bicarbonate followed by acetonitrile, then reduced with 10 mmol/L dithiothreitol at 60°C followed by alkylation with 50 mmol/L iodoacetamide at RT, digested with trypsin (Promega) at 37°C for 4 h, and finally quenched with formic acid. Peptides were lyophilized and reconstituted in 60 *μ*L of 0.1% TFA containing 0.4 fmols/*μ*L of each internal standard peptide (New England Peptide). Half of each digested sample was analyzed by nano LC‐PRM/MS with a Waters NanoAcquity HPLC system interfaced to a ThermoFisher Q Exactive HF. The mass spectrometer was operated in Parallel Reaction Monitoring (PRM) mode with the Orbitrap operating at 60,000 FWHM and 17,500 FWHM for MS and MS/MS respectively. Data were collected for the target peptides as outlined in Table 1. Final values were expressed as a mol/*μ*L of eluate.

**Table 1:** Target peptide sequences used in LC‐PRM/MS

Target protein	Peptide Sequence	Precursor ion *m/z*	Product ion *m/z*
AKT1	SLLSGLLK	415.7709	517.418
			630.418
	SLLSGLLK[HeavyK]	419.7780	525.348
			638.432
AKT2	SLLAGLLK	407.7735	501.339
			614.423
	SLLAGLLK[HeavyK]	411.7806	509.353
			622.437
AKT3	SLLSGLLIK	472.3130	630.418
			743.502
	SLLSGLLIK[HeavyK]	476.3201	638.432
			751.516

### Immunocytochemistry, microscopy, and image analysis

Cells were seeded and differentiated on collagen‐coated coverslips followed by fixation and staining exactly as previously described (Matheny et al. 2015). Images were captured using a Zeiss LSM700 confocal microscope and Zen analysis software. Results are presented as means ± standard errors from three independent experiments, with each experimental point derived from five randomly captured fields for each treatment group. Myotube fusion index was determined by counting the nuclei in every myotube (defined as MyHC‐positive cells containing a minimum of two nuclei) per field and dividing by the total number of nuclei in the field.

### Statistics

Data are presented as mean ± SEM. Statistics were performed using one‐way ANOVA with Dunnetts's multiple comparison test performed post hoc. A *P* < 0.05 was considered significant.

## Results

**Figure 1:**
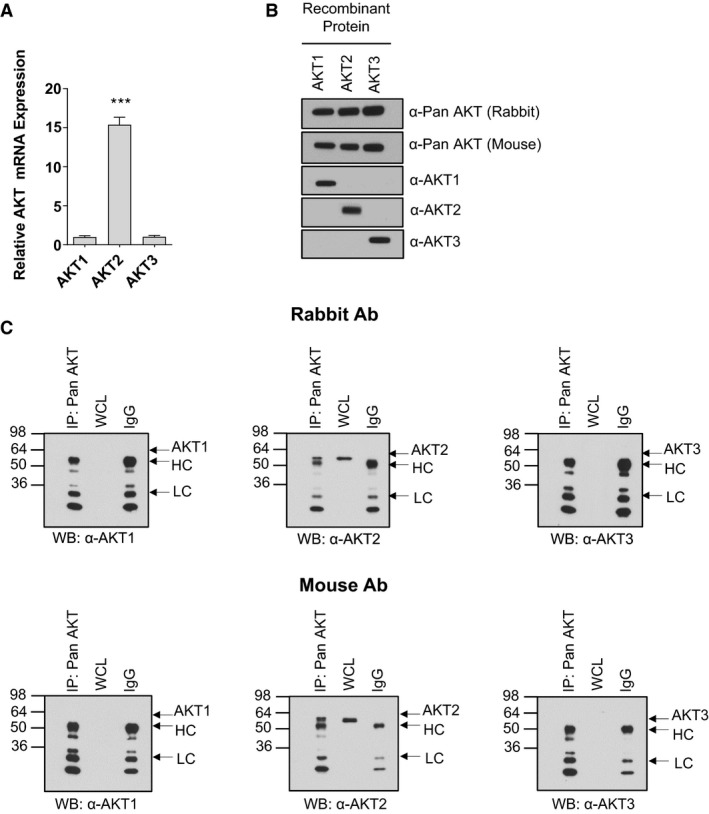
RNA transcript and protein expression of AKT isoforms in human skeletal muscle. Real‐time PCR was performed on cDNA derived from human vastus lateralis using AKT isoform‐specific primers/probes. Expression levels are normalized to expression of *AKT1* which was set to 1.0. (means ± SEMs; *n* = 4; ***, *P* < 0.001 versus *AKT1* and *AKT3*). (B) Western immunoblotting was performed on synthetic peptides of full‐length human AKT1, AKT2, or AKT3 using the indicated antibodies. (C) Immunoprecipitations were performed using either rabbit or mouse pan AKT antibodies followed by western blotting with isoform‐specific antibodies as indicated. Twenty‐five micrograms of whole cell lysate (“WCL”) were also subjected to SDS‐PAGE. Control immunoprecipitations were performed using the relevant species‐specific IgG instead of antibody. Molecular mass in kDa is shown on the left of each blot. “HC;” heavy chain; LC, light chain.

We first identified the RNA transcript expression of each AKT isoform in biopsies obtained from human vastus lateralis. We found that AKT2 was the most highly expressed AKT transcript, exhibiting a 15.4‐fold increase over AKT1 transcripts (Fig. 1A). Expression of AKT3 mRNA was similar to expression of AKT1.

Next, we examined whether this relationship between AKT isoform expressions was maintained at the protein level. Initial trials using LC‐MS/MS were able to detect only AKT2 protein in crude lysates from human vastus lateralis (data not shown). To confirm this finding, we subjected lysates to immunoprecipitation using total (pan) AKT antibodies (both rabbit and mouse species), followed by Western immunoblotting using antibodies raised against each individual AKT isoform. Two distinct pan AKT antibodies were separately used for immunoprecipitation to account for potential differences in affinity toward AKT isoforms and toward the protein A/G beads used for immunoprecipitation. Both rabbit and mouse pan AKT antibodies were immunoreactive with recombinant peptides of all three AKT isoforms, and antibodies for each AKT isoform recognized only the specific recombinant peptide for which it was expected to detect (Fig. 1B). Following immunoprecipitation using the rabbit and mouse pan AKT antibodies, only AKT2 was detectable by western blot (Fig. 1C). Longer exposures failed to detect either endogenous or pan AKT‐immunoprecipitated AKT1 or AKT3 (not shown). When the reciprocal experiment was performed (immunoprecipitation with isoform‐specific AKT antibodies followed by western blotting with pan AKT antibodies), AKT2 was again the only AKT isoform evident (data not shown).

**Figure 2:**
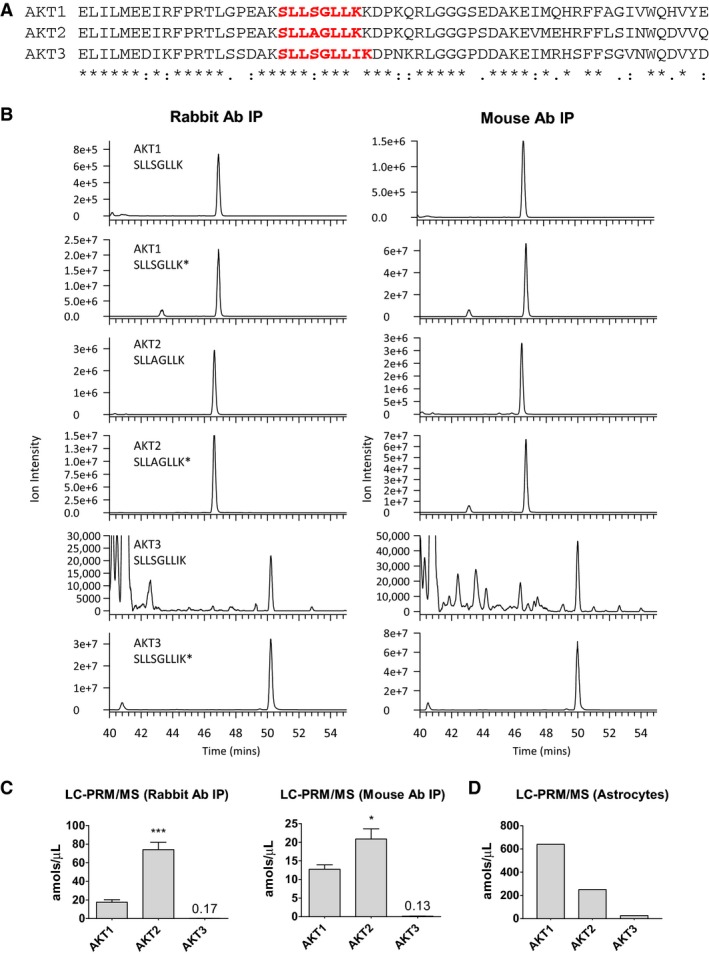
AKT isoform abundance in human skeletal muscle. (A) Amino acid sequence alignment among human AKT isoforms. Sequences represent residues 359–418 from AKT1 (NCBI Reference Sequence: NP_001014432.1), residues 360–419 from AKT2 (NCBI Reference Sequence: NP_001617.1), and residues 356–415 from AKT3 (NCBI Reference Sequence: NP_005456.1). Regions in red font indicate residues of peptide sequences used in LC‐PRM/MS. (B) Representative chromatograms for AKT isoforms using purified peptides and following immunoprecipitation of protein lysates using rabbit (left) and mouse (right) pan AKT antibodies. Asterisks denote the stable labeled synthetic internal standard peptide. (C) LC‐PRM/MS was performed on eluates derived from immunoprecipitations of human vastus lateralis lysates using either rabbit or mouse pan AKT antibodies, as indicated (means ± SEMs; *n* = 4 subjects; *, *P* < 0.05 versus AKT1 and AKT3; ***, *P* < 0.001 versus AKT1 and AKT3). “amol;” attomoles; “*μ*L;” microliter. (D) LC‐PRM/MS was performed on eluates derived from immunoprecipitations of human astrocyte lysates using rabbit pan AKT antibody.

We next performed immunoprecipitations using rabbit and mouse pan AKT antibodies followed by LC‐PRM/MS. Data were collected for each AKT isoform using unique target peptides and protein samples immunoprecipitated with pan AKT antibodies (Fig. 2A and B). Using this approach, we were able to detect all three AKT isoforms in human muscle lysate, identifying AKT2 as the most highly expressed AKT protein (4.2‐fold greater than AKT1 using the rabbit antibody and 1.6‐fold greater than AKT1 using the mouse antibody) (Fig. 2C). AKT3 was the least abundant AKT isoform, expressed at a level ~1% that of AKT1. As a technical control, we also performed LC‐PRM/MS on lysates derived from primary human astrocytes, as the distribution of AKT isoforms was expected to differ from that of muscle (Endersby et al. 2011). The relationship between AKT isoform abundances in astrocytes did indeed differ from that observed in muscle: AKT1 was the most abundant AKT isoform in astrocyte lysates, expressed 2.5‐fold greater than AKT2 and 22.9‐fold greater than AKT3 (Fig. 2D).

**Figure 3:**
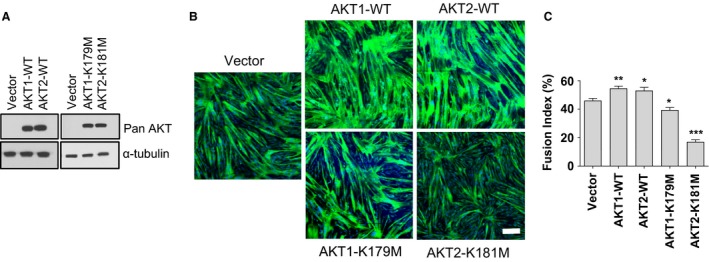
Expression of kinase‐inactive AKT2 markedly inhibits differentiation of primary human skeletal myoblasts. (A) Western blotting was performed on lysates derived from human skeletal myoblasts transduced with control vector (Vector), wild‐type AKT (WT‐AKT1 or WT‐AKT2), kinase‐inactive AKT1 (AKT1‐K179M), or kinase‐inactive AKT2 (AKT2‐K181M) using the indicated antibodies. (B) Human skeletal myoblasts were transduced with the indicated constructs and allowed to differentiate for 72 h before fixation and staining with MyHC (green) and DAPI (blue). (C) Quantification of myotube fusion index from experiments whose results are shown in panel A (means ± SEMs; *n* = 3 independent experiments; 5 fields analyzed per experimental point; *P < 0.05; **P < 0.01; ***P < 0.001. Scale bar: 200 *μ*m.

Given that Akt has been shown to play an important role in murine myoblast differentiation (Rotwein and Wilson 2009; Heron‐Milhavet et al. 2008), and having found that AKT2 is the most highly expressed AKT isoform in human quadriceps, we next tested whether AKT2 was the principal isoform regulating human myotube formation. Cultured primary human myoblasts were virally‐transduced with cDNAs encoding either wild‐type (WT) or kinase‐inactive AKT1 (K179M) or AKT2 (K181M) and allowed to terminally differentiate. Myotubes expressing either AKT1‐WT or AKT2‐WT showed enhanced fusion compared to control myotubes, and myotubes expressing AKT1‐K179M showed modestly reduced fusion index (14% less than control; *P* < 0.05); however, myotubes expressing AKT2‐K181M showed a dramatic decrease in fusion index (−63%; *P* < 0.001) compared to control myotubes (Fig. 3).

## Discussion

In this report, we provide quantitative data regarding the expression of AKT isoforms in human skeletal muscle. Our observations show that AKT2 is the most highly‐expressed AKT isoform, with several lines of experimental evidence supporting this finding. First, expression of AKT2 mRNA was >15‐fold higher than expression of AKT1 or AKT3 mRNA. Next, LC‐MS/MS was only able to detect AKT2 in crude protein lysates, suggesting that the abundance of AKT2 sufficiently exceeded the minimal level of detection for this method, whereas the abundances of AKT1 and AKT3 did not. Similarly, antibody immunoprecipitations using isoform‐specific and pan AKT antibodies were only able to detect AKT2 following Western immunoblotting. Finally, we were able to detect all three AKT isoforms using LC‐PRM/MS, and among them, AKT2 was the most abundant. This was the case whether protein lysates were immunoprecipitated with rabbit or mouse pan AKT antibodies that each recognized distinct epitopes. Together, these data demonstrate that AKT2 is the predominant AKT isoform expressed in human skeletal muscle.

We also found that AKT3 was barely detectable at the protein level despite an obvious expression of AKT3 mRNA. This was surprising, especially given our observation that expression of AKT3 mRNA was similar to the expression of AKT1 mRNA. Several possible posttranscriptional mechanisms may underlie this finding, including a decreased the rate of AKT3 mRNA nuclear export, decreased AKT3 protein synthesis, or increased degradation. Additionally, it has been reported that the correlation between mRNA and protein abundance is only ~40%, and that protein levels are primarily dependent on translational, rather than transcriptional, regulatory mechanisms (Schwanhausser et al. 2011). While the exact mechanism(s) regulating AKT3 expression in human skeletal muscle is unknown, the lack of AKT3 protein suggests that AKT3 plays little, if any, role in human skeletal muscle physiology or metabolism.

We observed that expression of kinase‐inactive AKT2 inhibited primary human myoblast differentiation to a much greater degree than expression of kinase‐inactive AKT1, thus quantifying the contributions of AKT isoforms in human myoblast differentiation. Our findings are in general agreement with previous studies that have reported significant roles for Akt2 in mediating rodent myoblast differentiation (Heron‐Milhavet et al. 2008; Vandromme et al. 2001). However, other studies have reported that Akt1 is the primary Akt isoform mediating murine myoblast differentiation, with Akt2 playing a much more limited role (Wilson and Rotwein 2007; Gardner et al. 2012). The discrepancies between these reports suggest that the actions of Akt isoforms in mediating myoblast differentiation may be species‐ and cell line‐specific. Nonetheless, our observations in differentiating primary human myoblasts demonstrate that the kinase activity of both AKT1 and AKT2 are necessary for full myoblast differentiation, and that the contribution of AKT2 activity is substantially greater than the contribution of AKT1 activity to this process.

While we did not interrogate specific molecular pathways through which kinase‐inactive AKT mutants may be exerting their negative effects on differentiation, AKT has previously been shown to regulate numerous molecules involved in myogenic differentiation in an isoform‐specific manner. For example, the basic helix‐loop‐helix transcription factor myogenin has long been recognized to promote myogenesis, and in insulin‐stimulated C2C12 murine myoblasts, Akt1 inhibited the transcriptional activity of myogenin, whereas Akt2 promoted myogenin transcription (Sumitani et al. 2002). Because the kinase activity of AKT is necessary for activation of downstream effector molecules, the reduced fusion we observed in differentiating myoblasts transduced with kinase‐inactive mutants likely resulted from altered AKT‐mediated downstream signaling.

The relatively high abundance of AKT2 in human skeletal muscle may suggest a degree of functional redundancy among AKT isoforms, and raises the possibility that skeletal muscle AKT2 assumes functional roles otherwise played by AKT1 or AKT3 in other tissues. However, the propensity for AKT2 to perform these roles would not be expected to depend solely on its abundance, as the activation of AKT is influenced by additional factors. Indeed, AKT activation is dependent on a variety of inputs such as localization to PI(3,4)P2 and PI(3,4,5)P3 in the cell membrane (Andjelkovic et al. 1997; Franke et al. 1997), autophosphorylation (Toker and Newton 2000), and dephosphorylation by isoform‐specific AKT phosphatases (Gao et al. 2005). These processes – independently and collectively – mediate activation, localization, and AKT‐mediated downstream signaling cascades. It is the extent to which these processes are balanced that ultimately regulate AKT activation in human skeletal muscle.

In summary, we found that among the three AKT isoforms in human skeletal muscle, AKT2 was the most highly‐expressed AKT isoform, AKT1 was the second‐most highly‐expressed AKT isoform, and AKT3 was the least‐expressed AKT isoform. Additionally, the kinase activity of AKT2 was principally necessary for the differentiation of primary human skeletal myoblasts. Defining the specific actions of AKT isoforms in human skeletal muscle continues to be an important step in identifying AKT‐mediated processes that contribute to skeletal muscle health and disease.

## Conflict of Interest

All authors declare no conflict of interest.
